# Right Hemicolectomy and Appendicectomy as Treatments for Goblet Cell Adenocarcinoma: A Comparative Analysis of Two Large National Databases

**DOI:** 10.3390/curroncol31070285

**Published:** 2024-07-02

**Authors:** Marie Line El Asmar, Mohamed Mortagy, Kandiah Chandrakumaran, Tom Cecil, John Ramage

**Affiliations:** 1 Department of Gastroenterology, Hampshire Hospitals NHS Foundation Trust, Basingstoke RG24 9NA, UK; john.ramage@hhft.nhs.uk; 2 Hampshire Hospitals NHS Foundation Trust, Winchester SO22 5DG, UK; mohamed.mortagy2@hhft.nhs.uk; 3 Internal Medicine Department, St. George University School of Medicine, West Indies, Grenada; 4 Peritoneal Malignancy Institute, Hampshire Hospitals NHS Foundation Trust, Basingstoke RG24 9NA, UK; kandiah.chandrakumaran@hhft.nhs.uk (K.C.); tom.cecil@hhft.nhs.uk (T.C.); 5 Faculty of Health and Wellbeing, University of Winchester, Hampshire SO22 4NR, UK

**Keywords:** goblet cell adenocarcinoma, survival, right hemicolectomy, appendicectomy, SEER, NCRAS

## Abstract

Introduction: Right hemicolectomy (RHC) remains the treatment standard for goblet cell adenocarcinoma (GCA), despite limited evidence supporting survival benefit. This study aims to explore factors influencing surgical management and survival outcomes among patients treated with RHC or appendicectomy using NCRAS (UK) and SEER (USA) data. Methods: A retrospective analysis was conducted using 998 (NCRAS) and 1703 (SEER) cases. Factors influencing procedure type were explored using logistic regression analyses. Overall survival (OS) probabilities and Kaplan–Meier (KM) plots were generated using KM analysis and the log-rank test compared survival between groups. Cox regression analyses were performed to assess hazard ratios. Results: The NCRAS analysis revealed that age and regional stage disease were determinants of undergoing RHC, with all age groups showing similar odds of receiving RHC, excluding the 75+ age group. The SEER analysis revealed tumour size > 2 cm, and receipt of chemotherapy were determinants of undergoing RHC, unlike the distant stage, which was associated with appendicectomy. Surgery type was not a significant predictor of OS in both analyses. In NCRAS, age and stage were significant predictors of OS. In SEER, age, stage, and Black race were significant predictors of worse OS. Conclusions: The study shows variations in the surgical management of GCA, with limited evidence to support a widespread recommendation for RHC.

## 1. Introduction

Goblet cell adenocarcinoma (GCA), arising from the appendix, is rare and comprises less than 15% of primary appendix tumours [1]. The World Health Organization (WHO) reclassified GCA as an adenocarcinoma, replacing the Tang et al. classification [2,3]. The presenting symptoms are typically vague, mimicking appendicitis [4,5,6]. Consequently, pre-operative diagnosis is rare, with diagnoses made incidentally post-appendicectomy or after ileocecal resection. However, patients may present symptomatically with advanced-stage disease [3,7,8,9].

Debate surrounds the surgical management of GCA. Consensus guidelines from the UK and Ireland Neuroendocrine Tumour Society (UKI NETS), North American Neuroendocrine Tumor Society (NANETS), and European Neuroendocrine Tumor Society (ENETS) recommend RHC within three months of the initial appendicectomy [7,10,11]. In fact, 20–40% of patients with primary GCA have lymph node involvement and undergo RHC [3,12]. However, for patients with peritoneal involvement, cytoreductive surgery (CRS) with heated intraperitoneal chemotherapy (HIPEC) may be offered, which is the standard of care for Pseudomyxoma Peritonei from an appendiceal primary [3,12,13,14]. In higher-grade tumours that were resected, lymph node metastases were infrequent compared to the trans-coelomic spread, suggesting that GCA may spread peritoneally [14,15,16]. There may be a case for CRS and HIPEC in GCA, although, currently, this is unproven [17,18].

Evidence on the benefit of RHC on survival outcomes is lacking, especially for small and low-grade tumours. RHC was shown to have potential survival benefits in advanced-stage tumours in a retrospective analysis [19]. However, completion surgery did not appear to improve recurrence or survival in some studies [20]. Ambiguous recommendations contribute to varying clinical practices, which raises concerns of patients being undertreated with appendicectomy or potentially overtreated with RHC, considering morbidity rises to 40% in elderly patients post-RHC [7].

This study, using data from the National Cancer Registry and Analysis Service (NCRAS) in the UK and the Surveillance, Epidemiology, and End Results (SEER) from the USA, compares survival outcomes between patients managed with RHC or appendicectomy alone. It also explores factors influencing the choice of surgery.

## 2. Methods

### 2.1. Study Design and Data Sources (NCRAS and SEER)

This was a population-based study using retrospective data of 998 patients (NCRAS) and 1703 patients (SEER). NCRAS collects prospective data of all diagnosed tumours within the NHS in England [21]. SEER, established in 1973, provides data on cancer statistics covering 50% of the US population [22].

### 2.2. Data Extraction

#### 2.2.1. NCRAS

A total of 1345 GCA cases occurring between 1995 and 2018 were extracted using the International Classification of Diseases for Oncology, 3rd edition (ICD-03), morphology code 8243 [23]. There were 1225 of appendiceal origin, after excluding 129 non-appendiceal GCA cases. The Office of Population Censuses and Surveys (OPCS-4) was used to classify surgeries [24] (Supplementary Table S1). A further 227 cases were excluded for missing or other surgical codes. The final analysis included 998 cases. TNM staging data, missing in over 50% of cases, was omitted. Tumour size and lymph node involvement data, with 90% missing, were also omitted. Stages 0, I, and II were combined into ‘Localised/In situ’, Stage III was categorised as ‘Regional’, and Stage IV as ‘Distant’ to match SEER data. Grade was omitted as the grade classification transitioned from Tang to WHO during data collection, resulting in inconsistent data.

#### 2.2.2. SEER

A total of 1884 cases of GCA were extracted from three databases of SEER by using the SEER∗Stat software, version 8.4.3 [25,26,27,28]. ICD-03 morphology code 8243 and the ICD-03 appendix site code C18.1 were used to extract these patients [24]. A total of 49 patients diagnosed from 1975 to 1991 were extracted from the database (Incidence—SEER Research Data, 8 Regs, Nov 2022 submission [1975–2020]), and 66 patients diagnosed from 1992 to 1999 were extracted from the database (Incidence—SEER Research Data, 12 Regs, Nov 2022 submission [1992–2020]). A total of 1769 patients diagnosed in 2000–2020 were extracted from the database (Incidence—SEER Research Data, 17 Regs, Nov 2022 submission [2000–2020]). The extracted cases were combined into one cohort, which maximised the sample size while avoiding an overlap of cases. Two patients were excluded for not having a positive histology or cytology diagnosis. Stage, tumour size, and procedure type were combined from the three databases (see Supplementary Table S2). SEER surgical codes 40 and 41 were used to identify RHC, and codes 20, 27, 29, 30, and 32 were used to identify appendicectomy. A further 179 cases were excluded for missing data or having other surgical codes. The final analysis included 1703 cases. A flowchart detailing the stages of data extraction and a table of variable formation are shown in Supplementary Figure S1 and Table S2, respectively. “Historical stage” was used instead of stages I–IV in the SEER analysis due to 24.9% missing values [26]. Grade was omitted from the analysis for having 83.5% missing data.

### 2.3. Data Analysis (NCRAS and SEER)

Descriptive data included categorical variables presented in frequencies and percentages and numerical variables presented with mean, median, and standard deviation. A Pearson’s chi-squared test evaluated the differences between the groups and a *t*-test evaluated the differences between the means. Univariable logistic regression analyses were performed to study factors associated with the choice of surgery. Multivariable logistic regression analyses included variables demonstrating statistical significance in the univariable logistic regression analyses.

Kaplan–Meier (KM) analysis was used to estimate overall survival probabilities at 1, 12, 36, and 60 months up to the date of death or last follow-up (censored) and to generate KM plots. The log-rank test compared survival plots between the two surgical groups, and a *p*-value of <0.05 was deemed statistically significant. Both survival analyses were relative survival analyses (not cancer-specific).

A univariable Cox regression analysis was performed and unadjusted hazard ratios (HRs) were generated with 95% confidence intervals. Statistically significant and clinically relevant variables in the univariable analyses were included in the multivariable Cox regressions. Variables with missing data or those that failed to converge were omitted from the multivariable models. Proportional hazard assumption tests were conducted on all multivariable Cox regression models. All statistical analyses were performed using Rstudio version 2023.12.0 Build 369.

## 3. Results 

### 3.1. Baseline Characteristics

#### 3.1.1. NCRAS

A total of 998 patients were included from NCRAS (Table 1). Among the 998 GCA cases, 71.0% (n = 710) underwent RHC and 29.0% (n = 289) underwent appendicectomy. The mean age was 58.7 years, with an almost equal gender distribution (49.5% male and 50.5% female). Localised/in situ accounted for 71.6% of the cohort, 75% of the RHC group, and 78.5% of the appendicectomy group, with nearly equal representation across. The majority were White (n = 933, 93.5%), exceeding the proportion in England’s population (according to 2021 consensus, 81.7% White) [29]. There was a uniform spread of Index of Multiple Deprivation (IMD) categories, with most from predominantly urban areas (Table 1). Statistically significant differences between the groups were observed in age and cancer stage. No significant differences were found in sex, rurality, ethnicity, or IMD (Table 1).

#### 3.1.2. SEER

A total of 1703 patients were included from SEER (Table 1). Among the cases, 53.0% (n = 909) underwent RHC, and 47.0% (n = 794) underwent appendicectomy. The mean age was 58.0 years, with an almost equal representation of males (52.0%) and females (48.0%). The majority were White (n = 1485, 87.2%), and 8.3% were Black, compared to that of the US population (75.5% and 13.9%, respectively) [30]. Localised/in situ stage represented 54.8% of the cohort. A total of 36.5% of tumours were less than 2 cm and 31.5% were more than 2 cm in size. Most patients did not receive chemotherapy (84.2%). Statistically significant differences between the two groups were observed in stage, tumour size, chemotherapy, and treatment delay. No significant differences were found in age, sex, race, or income.

Supplementary Figures S2A (NCRAS) and S2B (SEER) present the trends and distributions of surgery types per diagnosis year. From 2000 to 2018, the rate of RHC in England appears higher than that of appendicectomy. However, there is no consistent trend pattern for either procedure. In the US, both RHC and appendicectomy show a steady rise, with RHC slightly exceeding appendicectomy. The geographical distribution of GCA cases in England is shown in (Supplementary Table S3). A statistically significant difference between the uptake of RHC across different regions in England was found (*p* < 0.001). London and South East England had the highest and lowest uptake of RHC compared to other regions in England, respectively (Supplementary Table S4).

### 3.2. Factors Associated with Undergoing RHC versus Appendicectomy

#### 3.2.1. NCRAS

The multivariable analysis revealed that, compared to individuals aged 29 years or younger, those in the 30–54 (OR: 4.67, *p* = 0.002), 55–64 (OR: 3.97, *p* = 0.006), and 65–74 (OR: 4.05, *p* = 0.006) years age groups were roughly four times more likely to undergo RHC. However, there was a non-significant association for the 75+ years age group (OR: 1.95, *p* = 0.199). Patients with regional stage disease were more likely to receive RHC (OR: 3.20, *p* = 0.002), compared to localised/in situ stage disease, while distant stage appeared to have a non-significant association (OR: 1.94, *p* = 0.066) (Table 2).

#### 3.2.2. SEER

The multivariable analysis revealed tumour size > 2 cm (OR: 1.83, *p* ≤ 0.001) and having received chemotherapy treatment (OR: 2.14, *p* = 0.001) were independent factors associated with receiving RHC. Distant stage was associated with receiving appendicectomy (OR: 0.49, *p* = 0.016).

Regional stage, treatment delay, and USD 60,000–70,000 income were associated with increased odds of receiving RHC only in the univariable analysis (Table 3).

### 3.3. Kaplan–Meier Survival Analysis

**Figure 1:**
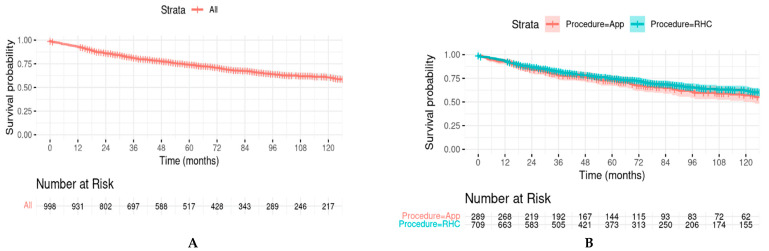

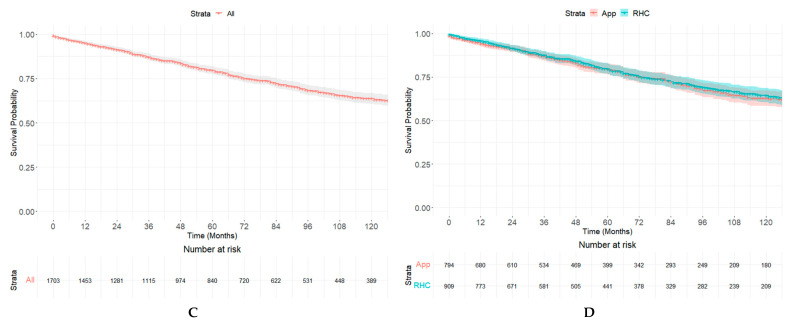
(**A**) Kaplan–Meier curves showing overall survival probabilities of GCA (NCRAS); (**B**) Kaplan–Meier curves showing survival probabilities of GCA by procedure type (NCRAS); (**C**) Kaplan–Meier curves showing overall survival probabilities of GCA (SEER); (**D**) Kaplan–Meier curves showing survival probabilities of GCA by procedure type (SEER). Log-rank test *p*-value (NCRAS) for the difference between appendicectomy and RHC = 0.2; log-rank test *p*-value (SEER) for the difference between appendicectomy and RHC = 0.3.

The estimated overall survival (OS) rates from NCRAS at 1 month, 12 months, 36 months, and 60 months were 98.0%, 92.8%, 81.0%, and 73.8%, respectively (Supplementary Table S5). The estimated OS rates from SEER at 1 month, 12 months, 36 months, and 60 months were 98.6%, 95.0%, 87.2%, and 79.6%, respectively (Supplementary Table S5). Figure 1A,C represent KM plots of overall survival for the NCRAS and SEER cohorts, respectively. KM plots comparing the survival of patients receiving RHC (green curves) or appendicectomy (red curves) showed no significant differences in survival curves between the groups on the log-rank test (*p* = 0.2 NCRAS, *p* = 0.3 SEER) (B,D).

#### 3.3.1. NCRAS Cox Regression Analyses

In the univariable Cox regression analyses, female sex (HR 1.27, *p* = 0.028), regional stage (HR 2.05, *p* ≤ 0.001), distant stage (HR 7.48, *p* ≤ 0.001) disease, and residing in most deprived areas (HR 1.48, *p* = 0.026) were significant predictors of worse OS compared to their respective reference categories (Table 4). Age also appeared (HR 1.05, *p* < 0.001), indicating a 5% increase in the hazard of death for each additional year of age.

In the multivariable Cox regression analyses, age maintained the same significant effect (HR 1.05, *p* < 0.001). Regional stage (HR 2.07, *p* < 0.001) and distant stage (HR 6.98, *p* < 0.001) of disease remained significant predictors of OS. There was no significant difference in survival between patients who underwent RHC and appendicectomy in both univariable and multivariable models (*p* = 0.228 and *p* = 0.164, respectively) (Table 4). The proportional hazard assumption test showed no violations of assumptions.

#### 3.3.2. SEER Cox Regression Analyses

In the univariable Cox regression analyses, age appeared (HR 1.04, *p* < 0.001), indicating a 4% increase in the hazard of death for each additional year of age. Regional stage (HR 1.32, *p* = 0.007), distant stage (HR 10.20, *p* < 0.001), tumour size > 2 cm (HR 1.64, *p* < 0.001), chemotherapy-treated (HR 2.82, *p* < 0.001), treatment delay (HR 1.27, *p* = 0.001), and Black race (HR 1.55, *p* = 0.002) were significant predictors of worse OS compared to their respective reference categories (Table 5).

In the multivariable Cox regression analyses, age (HR 1.04, *p* < 0.001), regional stage (HR 1.45, *p* = 0.009), distant stage (HR 10.108, *p* < 0.001), and Black race (HR 1.65, *p* = 0.012) remained significant prognostic factors for worse OS. Procedure type was not a significant predictor of OS on either univariable and multivariable models (*p* = 0.344 and *p* = 0.166, respectively) (Table 5). Tumour size, chemotherapy, and treatment delay became non-significant in the multivariable model. The proportional hazard assumption test showed no violations of assumptions.

## 4. Discussion

### 4.1. Choice of Surgery

The NCRAS analysis revealed age as a significant determinant of undergoing RHC, with all age groups showing similar odds of receiving RHC except for the 75+ years group. Stage, particularly regional stage (stage III), emerged as a significant factor associated with RHC. Conversely, age did not appear to be a determinant of RHC in the SEER analysis. Instead, tumour size > 2 cm and receipt of chemotherapy influenced treatment with RHC, unlike distant stage, which was associated with receiving appendicectomy.

Given the rarity of GCA and the lack of prospective studies, there is no clear consensus on its management. Some propose that early-stage localised low-grade tumours measuring less than 1 cm with low proliferation rates, T1/T2 stage, negative appendiceal margins, and low rates of lymph node metastases could be managed with appendicectomy [4,9,31,32,33,34,35]. This is attributed to the low risk of spread, particularly in cases where the tumours present as appendicitis [36,37,38]. On the other hand, some argue that RHC should be performed for all cases, irrespective of pathological characteristics [7,10,39,40]. Others recommend opting for RHC when the tumour exhibits poor differentiation, presents atypical features histologically, involves the base of the appendix, demonstrates nodal metastasis, has a high mitotic count index, or if tumour size exceeds 2 cm, as the risk of metastases is greater [9,33,36,41,42,43,44].

### 4.2. Survival Analysis

Comparing NCRAS and SEER survival analyses, procedure type did not emerge as a significant predictor of OS in both analyses. In NCRAS, age and stage were significant predictors of OS. In SEER, age, stage and Black race were significant predictors of worse OS. One study showed favourable survival outcomes for White race in the US, consistent with our findings [45]. Age and stage have been established as independent prognostic factors for survival in GCA [8,45,46,47,48,49]. Notably, tumour size was not a significant predictor of OS, which is also consistent with previous findings [46,48].

Studies comparing survival benefits between RHC and appendicectomy in GCA remain limited. A SEER analysis from data collected between 1973 and 2011 similarly revealed no significant difference in survival for GCA patients based on surgery [50]. A meta-analysis in 2004 also suggested no survival benefit of RHC in localised low-grade disease with no caecal involvement [31].

Evidence suggests that RHC may not be universally beneficial. Several retrospective studies have failed to demonstrate a conclusive benefit of RHC compared to appendicectomy, especially in early-stage disease [9,19,51]. A study using data from the National Cancer Database (NCDB) in the US found that approximately 60–70% of patients with appendiceal GCA had RHC, which is a relatively steady rate in recent years. Conversely, about 40% of patients with appendiceal NEN underwent RHC, with this rate declining over time with a shift toward appendicectomy [19]. The NCRAS and SEER data used in this study showed that RHC rates were higher in the UK than in the US (71% versus 53%). This large difference is unexplained, but could be related to healthcare system differences. The different timescales of data collection may be also a factor contributing to the differences in rates. Traditionally, the rationale behind RHC for GCA has been the removal of regional lymph nodes to prevent metastatic spread [3]. However, recent evidence suggests that peritoneal spread may be the pathway for GCA spread rather than lymph node involvement for high-grade pathology at appendicectomy [16]. With peritoneal spread being more likely, CRS/HIPEC may be a more logical therapy for high-risk cases, as it involves removing visible tumours surgically and bathing the abdominal cavity in heated chemotherapy [52]. Further evidence is needed to compare the survival outcomes of the different surgical approaches.

### 4.3. Implications of These Data for Guidelines

Given the large variability in practice and recommendations, evidence must be gathered to make firm recommendations for surgical management. There were no clear factors that appeared to mandate RHC, although there were some associations with age and stage. The findings in this study from two large databases showed no clear survival benefit of RHC over appendicectomy. However, it remains uncertain what the survival outcomes would have been if patients who underwent RHC had not opted for it. The findings also suggest that decisions on RHC in this condition are somewhat arbitrary, indicating a possible randomised allocation to procedures. The optimal understanding of RHC’s role would be best attained through prospective studies. However, given the rarity of GCA, conducting such studies is unlikely due to logistical constraints.

These findings could impact recommendations, potentially requiring guideline revisions. Currently, neuroendocrine tumour societies offer guidelines, but ideally, colorectal surgical societies in conjunction with colorectal centres would develop them. For high-risk cases, peritoneal surgery centres could be consulted to determine the suitability of CRS and HIPEC. Long-term follow-up is necessary in most cases, and guidelines need to be available for this purpose.

### 4.4. Strengths and Limitations

This study represents the largest sample size (2700 patients) on GCA. The comparative analysis also potentially reveals insights into geographical variations and healthcare influences. The data represent Western populations with limited ethnic diversity, affecting generalisability. There were >80% missing data in tumour size and lymph node involvement in NCRAS, affecting data completeness. Data on adjuvant treatments (chemotherapy and radiotherapy) were deemed inaccurate in NCRAS and were omitted, which are potential unmeasured confounders. The lack of a cause of death in NCRAS may be of some concern, yet debates persist around the accuracy of cancer-specific mortality [53]. Some variables in SEER, like chemotherapy and treatment delay, have limitations due to incompleteness and imprecision [54]. Localised/in situ stage disease represented 71.6% and 54.8% of the NCRAS and SEER cohorts, respectively. The relatively high percentage of localised disease may have affected the outcome of the study, causing procedure type to remain an insignificant predictor of OS. However, this finding may suggest that early-stage disease could be managed with appendicectomy alone. Other confounders, not recorded in the registries, may have affected surgical choice, such as patient health status and other comorbidities, which should not add a systematic bias to the results.

## 5. Conclusions

The findings reveal a significant variability in the surgical approach to GCA. Although recommendations are for RHC, there is limited evidence for this. RHC may be beneficial for cases with enlarged local lymph nodes, implying local lymphatic spread. Small and low-grade GCS can possibly be treated with appendicectomy alone, followed by appropriate follow-up care. For higher-risk tumours, it is suggested that referral to a peritoneal cancer unit may be appropriate to consider CRS/HIPEC.

## Figures and Tables

**Table 1:** Baseline characteristics of the right hemicolectomy and appendicectomy cohorts (NCRAS and SEER).

	Overall	RHC	Appendicectomy	*p*-Value
	N (%)	N (%)	N (%)	
Total	998 (100%)	709 (71%)	289 (29%)	
NCRAS				
Age				<0.001 *
1 (≤29 years)	23 (2.3%)	8 (1.1%)	15 (5.19%)	
2 (30–54 years)	355 (35.6%)	267 (37.7%)	88 (30.45%)	
3 (55–64 years)	258 (25.9%)	190 (26.8%)	68 (23.53%)	
4 (65–74 years)	232 (23.2%)	170 (24%)	62 (21.45%)	
5 (75+ years)	130 (13%)	74 (10.4%)	56 (19.38%)	
Sex				0.605
Male	494 (49.5%)	355 (50.1%)	139 (48.1%)	
Female	504 (50.5%)	354 (49.9%)	150 (51.9%)	
Stage				<0.001 *
Localised/In situ	759 (71.6%)	532 (75%)	227 (78.5%)	
Regional	77 (7.7%)	68 (9.6%)	9 (3.1%)	
Distant	57 (5.7%)	47 (6.6%)	10 (3.5%)	
Unknown **	105 (10.5%)	62 (8.7%)	43 14.9%)	
Rurality				0.492
Predominantly Rural	100 (10.0%)	66 (9.3%)	34 (11.7%)	
Predominantly Urban	811 (81.3%)	580 (81.8%)	231 (79.9%)	
Urban with Significant Rural	87 (8.7%)	63 (8.9%)	24 (8.4%)	
Ethnicity				0.456
White	933 (93.5%)	668 (94.2%)	265 (91.7%)	
Asian	15 (1.5%)	12 (1.7%)	3 (1.0%)	
Black	10 (1%)	6 (0.8%)	4 (1.4%)	
Mixed race	4(0.4%)	3 (0.4%)	1 (0.4%)	
Other	5 (0.5%)	2 (0.3%)	3 (1.0%)	
Not stated **	31 (3.1%)	18 (2.5%)	13 (4.5%)	
IMD				0.151
1—Least deprived	189 (18.9%)	125 (17.6%)	64 (22.2)	
2	214 (21.4%)	160 (22.6%)	54 (18.7%)	
3	224 (22.4%)	164 (23.15%)	60 (20.8%)	
4	185 (18.5%)	123 (17.3%)	62 (21.4%)	
5—Most deprived	186 (18.6%)	137 (19.3%)	49 (16.9%)	
SEER				
	**Overall**	**RHC**	**Appendicectomy**	***p*** **-Value**
	**N (%)**	**N (%)**	**N (%)**	
**Total**	**1703 (100%)**	**909 (53%)**	**794 (47%)**	
Age				0.523
≤29 years	23 (1.4%)	8 (0.9%)	15 (1.9%)	
30–54 years	661 (38.8%)	370 (40.7%)	291 (36.6%)	
55–64 years	484 (28.4%)	248 (27.3%)	236 (29.7%)	
65–74 years	344 (20.2%)	188 (20.7%)	156 (19.6%)	
75+ years	191 (11.2%)	95 (10.5%)	96 (12.1%)	
Sex				0.839
Male	886 (52.0%)	479 (52.7%)	407 (51.3%)	
Female	817 (48.0%)	430 (47.3%)	387 (48.7%)	
Stage				<0.001 *
Localised/In situ	933 (54.8%)	453 (49.8%)	480 (60.5%)	
Regional	144 (8.5%)	81 (8.9%)	63 (7.9%)	
Distant	608 (35.7%)	368 (40.5%)	240 (30.2%)	
Unknown **	18 (1.1%)	7 (0.8%)	11 (1.4%)	
Tumour size (cm)				<0.001 *
<2	622 (36.5%)	286 (31.5%)	336 (42.3%)	
>2	537 (31.5%)	342 (37.6%)	195 (24.6%)	
Unknown **	544 (31.9%)	281 (30.9%)	263 (33.1%)	
Chemotherapy				<0.001 *
No/Unknown	1434 (84.2%)	726 (79.9%)	708 (89.2%)	
Yes	269 (15.8%)	183 (20.1%)	86 (10.8%)	
Treatment delay (months)				0.011 *
Mean (SD)	2.09 (0.462)	2.11 (0.514)	2.06 (0.392)	
Median [Min, Max]	2.00 [1.00, 9.00]	2.00 [1.00, 9.00]	2.00 [1.00, 7.00]	
Race				0.973
White	1485 (87.2%)	794 (87.3%)	691 (87.0%)	
Black	141 (8.3%)	74 (8.1%)	67 (8.4%)	
Other	66 (3.9%)	35 (3.9%)	31 (3.9%)	
Unknown **	11 (0.6%)	6 (0.7%)	5 (0.6%)	
Income				0.520
>USD 75,000	758 (44.5%)	420 (46.2%)	338 (42.6%)	
USD 40,000–USD 50,000	103 (6.0%)	51 (5.6%)	52 (6.5%)	
USD 50,000–USD 60,000	243 (14.3%)	134 (14.7%)	109 (13.7%)	
USD 60,000–USD 70,000	356 (20.9%)	174 (19.1%)	182 (22.9%)	
<USD 40,000	35 (2.1%)	19 (2.1%)	16 (2.0%)	
Unknown **	208 (12.2%)	111 (12.2%)	97 (12.2%)	

* Statistically significant; ** Excluded from the Pearson’s chi-squared test analysis.

**Table 2:** Univariable and multivariable logistic regression analyses (NCRAS).

	Univariable Logistic Regression			Multivariable Logistic Regression		
	OR	95%CI	*p*-Value	OR	95%CI	*p*-Value
Age						
1 (≤29 years)	1 (ref)					
2 (30–54 years)	5.69	2.39–14.55	<0.001 *	4.67	1.78–13.11	0.002 *
3 (55–64 years)	5.24	2.18–13.53	<0.001 *	3.97	1.49–11.28	0.006 *
4 (65–74 years)	5.14	2.13–13.33	<0.001 *	4.05	1.52–11.55	0.006 *
5 (75+ years)	2.48	1.00–6.54	0.054	1.95	0.71–5.68	0.199
Sex						
Male	1 (ref)					
Female	0.92	0.70–1.21	0.572			
Stage						
Localised/In Situ	1 (ref)			1 (ref)		
Regional	3.22	1.66–7.03	0.001 *	3.20	1.64–7.02	0.002 *
Distant	2.01	1.04–4.27	0.05	1.94	1.00–4.16	0.066
Unknown **						
Rurality						
Predominantly Rural	1 (ref)					
Predominantly Urban	1.29	0.82–2.00	0.253			
Urban with Significant Rural	1.35	0.73–2.55	0.345			
Ethnicity						
White	1 (ref)					
Asian	1.59	0.50–7.01	0.477			
Black	0.60	0.17–2.34	0.424			
Mixed race	1.19	0.15–24.12	0.880			
Other	0.26	0.03–1.60	0.146			
Not stated **						
IMD						
1—Least deprived	1 (ref)					
2	1.52	0.96–2.39	0.058			
3	1.40	1.04–2.60	0.119			
4	1.02	0.73–1.85	0.943			
5—Most deprived	1.43	0.92–2.33	0.113			

* Statistically significant; ** Excluded from the logistic regression analysis.

**Table 3:** Univariable and multivariable logistic regression analyses (SEER).

	Univariable Logistic Regression			Multivariable Logistic Regression		
	OR	95%CI	*p*-Value	OR	95%CI	*p*-Value
Age						
≤29 years	1 (ref)					
30–54 years	2.38	1.02–5.99	0.051			
55–64 years	1.97	0.84–4.97	0.129			
65–74 years	2.26	0.96–5.74	0.071			
75+ years	1.86	0.77–4.80	0.180			
Sex						
Male	1 (ref)					
Female	0.94	0.78–1.14	0.554			
Stage						
Localised/In Situ	1 (ref)			1 (ref)		
Regional	1.62	1.32–2.00	<0.001 *	1.29	0.98–1.72	0.073
Distant	1.36	0.96–1.94	0.086	0.49	0.27–0.87	0.016 *
Unknown **						
Tumour size (cm)						
<2	1 (ref)			1 (ref)		
>2	2.06	1.63–2.61	<0.001 *	1.83	1.40–2.39	<0.001 *
Unknown **						
Chemotherapy						
No/Unknown	1 (ref)					
Yes	2.08	1.58–2.75	<0.001 *	2.14	1.40–3.35	0.001 *
Treatment delay (months)	1.34	1.07–1.74	0.017 *	1.24	0.92–1.74	0.184
Race						
White	1 (ref)					
Black	0.96	0.68–1.36	0.823			
Other	0.98	0.60–1.62	0.944			
Unknown **						
Income						
>USD 75,000	1 (ref)			1 (ref)		
USD 60,000–USD 70,000	0.77	0.60–0.99	0.042	0.74	0.54–1.01	0.056
USD 50,000–USD 60,000	0.99	0.74–1.32	0.942	1.01	0.71–1.44	0.953
USD 40,000–USD 50,000	0.79	0.52–1.19	0.260	0.64	0.38–1.07	0.090
<USD 40,000	0.96	0.48–1.91	0.896	0.65	0.25–1.63	0.359

* Statistically significant; ** Excluded from the logistic regression analysis.

**Table 4:** Univariable and multivariable Cox regression analyses (NCRAS).

	Univariable Cox Regression			Multivariable Cox Regression		
	HR	95%CI	*p*-Value	aHR	95%CI	*p*-Value
Procedure						
Appendicectomy	1 (ref)			1 (ref)		
RHC	0.87	0.69–1.09	0.228	0.83	0.64–1.08	0.164
Age	1.05	1.04–1.06	<0.001 *	1.05	1.04–1.06	<0.001 *
Sex						
Male	1 (ref)			1 (ref)		
Female	1.27	1.03–1.57	0.028 *	1.04	0.83–1.31	0.725
Stage						
Localised/In Situ	1 (ref)			1 (ref)		
Regional	2.05	1.36–3.07	<0.001 *	2.07	1.37–3.12	<0.001 *
Distant	7.48	5.42–10.31	<0.001 *	6.91	4.98–9.58	<0.001 *
Unknown **						
Rurality						
Predominantly Rural	1 (ref)					
Predominantly Urban	0.82	0.58–1.14	0.234			
Urban with Significant Rural	0.75	0.46–1.22	0.253			
Ethnicity						
White	1 (ref)					
Asian	0.57	0.18–1.78	0.336			
Black	0.92	0.23–3.70	0.908			
Mixed race	0.54	0.08–3.91	0.549			
Other	1.86	0.59–5.79	0.287			
Not stated **						
IMD						
1—Least deprived	1 (ref)			1 (ref)		
2	1.15	0.81–1.62	0.435	1.13	0.78–1.64	0.530
3	0.94	0.67–1.33	0.743	0.92	0.63–1.33	0.665
4	1.30	0.91–1.85	0.145	1.37	0.94–1.99	0.102
5—Most deprived	1.48	1.04–2.08	0.026 *	1.41	0.98–2.04	0.063

* Statistically significant; ** Excluded from the Cox regression analysis.

**Table 5:** Univariable and multivariable Cox regression analyses (SEER).

	Univariable Cox Regression			Multivariable Cox Regression		
	HR	95%CI	*p*-Value	aHR	95%CI	*p*-Value
Procedure						
Appendicectomy	1 (ref)					
RHC	0.92	0.77–1.10	0.344	0.84	0.66–1.07	0.166
Age	1.04	1.03–1.05	<0.001	1.04	1.03–1.05	<0.001 *
Sex						
Male	1 (ref)					
Female	1.04	0.87–1.24	0.682			
Stage						
Localised/In Situ	1 (ref)			1 (ref)		
Regional	1.32	1.08–1.62	0.007	1.45	1.10–1.92	0.009 *
Distant	10.20	7.94–13.20	<0.001	10.08	6.33–16.05	<0.001 *
Unknown **						
Tumour size (cm)						
<2	1 (ref)			1 (ref)		
>2	1.64	1.30–2.08	<0.001	1.29	1.00–1.66	0.048
Unknown **						
Chemotherapy						
No/Unknown	1 (ref)			1 (ref)		
Yes	2.82	2.26–3.51	<0.001	1.13	0.74–1.70	0.574
Treatment delay (months)	1.27	1.10–1.46	0.001	1.25	1.00–1.56	0.054
Race						
White	1 (ref)					
Black	1.55	1.16–2.05	0.003	1.65	1.11–2.45	0.012 *
Other	0.75	0.44–1.27	0.283	0.65	0.32–1.32	0.237
Unknown **						
Income						
>USD 75,000	1 (ref)					
USD 60,000–USD 70,000	0.90	0.71–1.15	0.398			
USD 50,000–USD 60,000	1.25	0.97–1.62	0.091			
USD 40,000–USD 50,000	0.84	0.54–1.30	0.439			
<USD 40,000	1.61	0.90–2.89	0.108			
Unknown *						

* Statistically significant; ** Excluded from the Cox regression analysis.

## Data Availability

The datasets supporting the conclusions of this article are NCRAS: DARS-NIC-656877-H3Z0P-v1.4, available upon applying through the Data Access Request Service (DARS) (https://digital.nhs.uk/services/data-access-request-service-dars (accessed on 25 February 2021)] which is administered by NHS England. In addition, the SEER datasets generated and analysed in this study are anonymised public patient data and are available in the U.S. SEER database. Ethics clearance and informed patient consent were not required as the study involved secondary data analysis. The SEER datasets are accessible for free by following [https://seer.cancer.gov/ (accessed on 17 December 2023)].

## Supplementary Materials

The following supporting information can be downloaded at: https://www.mdpi.com/article/10.3390/curroncol31070285/s1, Figure S1: Flowchart detailing stages of data extraction (SEER); Figure S2: A (top): Distribution of procedure types by year of diagnosis (NCRAS); B (bottom): Distribution of procedure types by year of diagnosis (SEER); Table S1: OPCS-4 codes used to screen for type of surgery; Table S2: Variable formation (SEER); Table S3: Distribution of GCA cases across geographical regions in England (1995-2018); Table S4: Uptake of right hemicolectomy procedures across geographical regions in England; Table S5: Overall survival at 1 month, 12 months, 36 months, and 60 months.

## References

[1] McCusker M.E., Coté T.R., Clegg L.X., Sobin L.H. (2002). Primary malignant neoplasms of the appendix: A population-based study from the surveillance, epidemiology and end-results program, 1973–1998. Cancer.

[2] Nagtegaal I.D., Odze R.D., Klimstra D., Paradis V., Rugge M., Schirmacher P., Washington K.M., Carneiro F., Cree I.A. (2020). The 2019 WHO classification of tumours of the digestive system. Histopathology.

[3] Tang L.H., Shia J., Soslow R.A., Dhall D., Wong W.D., O’Reilly E., Qin J., Paty P., Weiser M.R., Guillem J. (2008). Pathologic classification and clinical behavior of the spectrum of goblet cell carcinoid tumors of the appendix. Am. J. Surg. Pathol..

[4] Park K., Blessing K., Kerr K., Chetty U., Gilmour H. (1990). Goblet cell carcinoid of the appendix. Gut.

[5] Shenoy S. (2016). Goblet cell carcinoids of the appendix: Tumor biology, mutations and management strategies. World J. Gastrointest. Surg..

[6] Kelly K.J. (2015). Management of appendix cancer. Clin. Colon Rectal Surg..

[7] Pape U.-F., Perren A., Niederle B., Gross D., Gress T., Costa F., Arnold R., Denecke T., Plöckinger U., Salazar R. (2012). ENETS Consensus Guidelines for the management of patients with neuroendocrine neoplasms from the jejuno-ileum and the appendix including goblet cell carcinomas. Neuroendocrinology.

[8] Lamarca A., Nonaka D., Lopez Escola C., Hubner R.A., O’Dwyer S., Chakrabarty B., Fulford P., Valle J.W. (2016). Appendiceal goblet cell carcinoids: Management considerations from a reference peritoneal tumour service centre and ENETS centre of excellence. Neuroendocrinology.

[9] Pham T.H., Wolff B., Abraham S.C., Drelichman E. (2006). Surgical and chemotherapy treatment outcomes of goblet cell carcinoid: A tertiary cancer center experience. Ann. Surg. Oncol..

[10] Boudreaux J.P., Klimstra D.S., Hassan M.M., Woltering E.A., Jensen R.T., Goldsmith S.J., Nutting C., Bushnell D.L., Caplin M.E., Yao J.C. (2010). The NANETS consensus guideline for the diagnosis and management of neuroendocrine tumors: Well-differentiated neuroendocrine tumors of the jejunum, ileum, appendix, and cecum. Pancreas.

[11] UKINETS (2016). UKINETS Bitesize Guidance on Goblet Cell Adenocarcinoma.

[12] Turaga K.K., Pappas S.G., Gamblin T.C. (2012). Importance of histologic subtype in the staging of appendiceal tumors. Ann. Surg. Oncol..

[13] Plöckinger U., Couvelard A., Falconi M., Sundin A., Salazar R., Christ E., De Herder W.W., Gross D., Knapp W.H., Knigge U.P. (2007). Consensus guidelines for the management of patients with digestive neuroendocrine tumours: Well-differentiated tumour/carcinoma of the appendix and goblet cell carcinoma. Neuroendocrinology.

[14] McConnell Y.J., Mack L.A., Gui X., Carr N.J., Sideris L., Temple W.J., Dubé P., Chandrakumaran K., Moran B.J., Cecil T.D. (2014). Cytoreductive surgery with hyperthermic intraperitoneal chemotherapy: An emerging treatment option for advanced goblet cell tumors of the appendix. Ann. Surg. Oncol..

[15] Clift A.K., Kornasiewicz O., Drymousis P., Faiz O., Wasan H.S., Kinross J.M., Cecil T., Frilling A. (2018). Goblet cell carcinomas of the appendix: Rare but aggressive neoplasms with challenging management. Endocr. Connect..

[16] Mehta A., Mittal R., Chandrakumaran K., Carr N., Dayal S., Mohamed F., Moran B., Cecil T. (2017). Peritoneal involvement is more common than nodal involvement in patients with high-grade appendix tumors who are undergoing prophylactic cytoreductive surgery and hyperthermic intraperitoneal chemotherapy. Dis. Colon Rectum.

[17] Shaib W.L., Martin L.K., Choi M., Chen Z., Krishna K., Kim S., Brutcher E., Staley III C., Maithel S.K., Philip P. (2015). Hyperthermic intraperitoneal chemotherapy following cytoreductive surgery improves outcome in patients with primary appendiceal mucinous adenocarcinoma: A pooled analysis from three tertiary care centers. Oncologist.

[18] Chua T.C., Moran B.J., Sugarbaker P.H., Levine E.A., Glehen O., Gilly F.N., Baratti D., Deraco M., Elias D., Sardi A. (2012). Early-and long-term outcome data of patients with pseudomyxoma peritonei from appendiceal origin treated by a strategy of cytoreductive surgery and hyperthermic intraperitoneal chemotherapy. J. Clin. Oncol..

[19] Marks V.A., Kerekes D., Butensky S., Ahuja N., Johnson C., Turaga K., Khan S.A. (2023). Role of colectomy in the management of appendiceal tumors: A retrospective cohort study. BMC Gastroenterol..

[20] Alabraba E., Pritchard D.M., Griffin R., Diaz-Nieto R., Banks M., Cuthbertson D.J., Fenwick S. (2021). Appendiceal goblet cell carcinomas have poor survival despite completion surgery. Endocrine.

[21] Cancer—NDRS. https://digital.nhs.uk/ndrs/about/ncras.

[22] NcicancerStats About the SEER Program-SEER. https://seer.cancer.gov/about/overview.html.

[23] Organisation W.H. International Classification of Diseases for Oncology, 3rd Edition (ICD-O-3). https://www.who.int/standards/classifications/other-classifications/international-classification-of-diseases-for-oncology.

[24] NHS Digital T.D.P. Classifications Browser. https://classbrowser.nhs.uk/#/book/OPCS-4.10/volume1-p2-3.html+P2_CHAPTER_H.

[25] NcicancerStats SEER*Stat Software. https://seer.cancer.gov/seerstat/index.html.

[26] National Cancer Institute, DCCPS, Surveillance Research Program (2023). SEER*Stat Database: Incidence—SEER Research Data, 8 Registries, Nov 2022 Sub (1975–2020)—Linked to County Attributes—Time Dependent (1990–2021) Income/Rurality, 1969–2021 Counties.

[27] National Cancer Institute, DCCPS, Surveillance Research Program (2023). SEER*Stat Database: Incidence—SEER Research Data, 12 Registries, Nov 2022 Sub (1992–2020)—Linked to County Attributes—Time Dependent (1990–2021) Income/Rurality, 1969–2021 Counties.

[28] National Cancer Institute, DCCPS, Surveillance Research Program (2023). SEER*Stat Database: Incidence—SEER Research Data, 17 Registries, Nov 2022 Sub (2000–2020)—Linked to County Attributes—Time Dependent (1990–2021) Income/Rurality, 1969–2021 Counties.

[29] GOV.UK Population of England and Wales. https://www.ethnicity-facts-figures.service.gov.uk/uk-population-by-ethnicity/national-and-regional-populations/population-of-england-and-wales/latest/.

[30] Bureau U.S.C. Quick Facts Unites States. https://www.census.gov/quickfacts/fact/table/US/PST045222.

[31] Varisco B., McAlvin B., Dias J., Franga D. (2004). Adenocarcinoid of the appendix: Is right hemicolectomy necessary? A meta-analysis of retrospective chart reviews. Am. Surg..

[32] Deans G., Spence R. (1995). Neoplastic lesions of the appendix. Br. J. Surg..

[33] Bucher P., Gervaz P., Ris F., Oulhaci W., Egger J.F., Morel P. (2005). Surgical treatment of appendiceal adenocarcinoid (goblet cell carcinoid). World J. Surg..

[34] Kowalsky S.J., Nassour I., AlMasri S., Paniccia A., Zureikat A.H., Choudry H.A., Pingpank J.F. (2021). Omission of right hemicolectomy may be safe for some Appendiceal goblet cell adenocarcinomas: A survival analysis of the National Cancer Database. Ann. Surg. Oncol..

[35] Edmonds P., Merino M.J., LiVolsi V.A., Duray P.H. (1984). Adenocarcinoid (mucinous carcinoid) of the appendix. Gastroenterology.

[36] Byrn J.C., Wang J.L., Divino C.M., Nguyen S.Q., Warner R.R. (2006). Management of goblet cell carcinoid. J. Surg. Oncol..

[37] Olsen I.H., Holt N., Langer S.W., Hasselby J.P., Grønbæk H., Hillingsø J., Mahmoud M., Ladekarl M., Iversen L.H., Kjaer A. (2015). Goblet cell carcinoids: Characteristics of a Danish cohort of 83 patients. PLoS ONE.

[38] Tsang E.S., McConnell Y.J., Schaeffer D.F., Lee L., Yin Y., Zerhouni S., Schaff K., Speers C., Kennecke H.F. (2018). Outcomes of surgical and chemotherapeutic treatments of goblet cell carcinoid tumors of the appendix. Ann. Surg. Oncol..

[39] Pericleous M., Lumgair H., Baneke A., Morgan-Rowe L., Caplin M.E., Luong T.V., Thirlwell C., Gillmore R., Toumpanakis C. (2012). Appendiceal goblet cell carcinoid tumour: A case of unexpected lung metastasis. Case Rep. Oncol..

[40] Gouzi J.-L., Laigneau P., Delalande J.-P., Flamant Y., Bloom E., Oberlin P., Fingerhut A. (1993). Indications for right hemicolectomy in carcinoid tumors of the appendix. The French Associations for Surgical Research. Surg. Gynecol. Obstet..

[41] Goede A., Caplin M., Winslet M. (2003). Carcinoid tumour of the appendix. J. Br. Surg..

[42] Pahlavan P.S., Kanthan R. (2005). Goblet cell carcinoid of the appendix. World J. Surg. Oncol..

[43] Butler J.A., Houshiar A., Lin F., Wilson S.E. (1994). Goblet cell carcinoid of the appendix. Am. J. Surg..

[44] Epithelial Tumors of the Appendix-UpToDate. https://www.uptodate.com/contents/epithelial-tumors-of-the-appendix?search=Appendiceal%20adenocarcinoma%20cancer%20stage&usage_type=default&source=search_result&selectedTitle=1~9&display_rank=1#H3104988092.

[45] Wang G., Li Q., Chen W. (2021). Chemotherapy in the treatment of different histological types of appendiceal cancers: A SEER based study. BMC Cancer.

[46] Fields A.C., Lu P., Enzinger A., Goldberg J., Irani J., Bleday R., Nash G., Melnitchouk N. (2019). Treatment patterns and outcomes in goblet cell carcinoid tumors of the appendix. J. Surg. Oncol..

[47] Yozu M., Johncilla M.E., Srivastava A., Ryan D.P., Cusack J.C., Doyle L., Setia N., Yang M., Lauwers G.Y., Odze R.D. (2018). Histologic and outcome study supports reclassifying appendiceal goblet cell carcinoids as goblet cell adenocarcinomas, and grading and staging similarly to colonic adenocarcinomas. Am. J. Surg. Pathol..

[48] Nonaka D., Papaxoinis G., Lamarca A., Fulford P., Valle J., Chakrabarty B. (2018). A study of appendiceal crypt cell adenocarcinoma (so-called goblet cell carcinoid and its related adenocarcinoma). Hum. Pathol..

[49] Taggart M.W., Abraham S.C., Overman M.J., Mansfield P.F., Rashid A. (2015). Goblet cell carcinoid tumor, mixed goblet cell carcinoid-adenocarcinoma, and adenocarcinoma of the appendix: Comparison of clinicopathologic features and prognosis. Arch. Pathol. Lab. Med..

[50] Shaib W., Krishna K., Kim S., Goodman M., Rock J., Chen Z., Brutcher E., Staley C.I., Maithel S.K., Abdel-Missih S. (2016). Appendiceal neuroendocrine, goblet and signet-ring cell tumors: A spectrum of diseases with different patterns of presentation and outcome. Cancer Res. Treat. Off. J. Korean Cancer Assoc..

[51] Fornaro R., Frascio M., Sticchi C., De Salvo L., Stabilini C., Mandolfino F., Ricci B., Gianetta E. (2007). Appendectomy or right hemicolectomy in the treatment of appendiceal carcinoid tumors?. Tumori J..

[52] Neuwirth M.G., Alexander H.R., Karakousis G.C. (2016). Then and now: Cytoreductive surgery with hyperthermic intraperitoneal chemotherapy (HIPEC), a historical perspective. J. Gastrointest. Oncol..

[53] Wong K.F., Lambert P.C., Mozumder S.I., Broggio J., Rutherford M.J. (2019). Conditional crude probabilities of death for English cancer patients. Br. J. Cancer.

[54] Noone A.-M., Lund J.L., Mariotto A., Cronin K., McNeel T., Deapen D., Warren J.L. (2016). Comparison of SEER treatment data with Medicare claims. Med. Care.

