# Supplementary material for: Right Hemicolectomy and Appendicectomy as Treatments for Goblet Cell Adenocarcinoma: A Comparative Analysis of Two Large National Databases
Source: Curr Oncol. 2024 Jul 2;31(7):3855–69. doi: 10.3390/curroncol31070285 (PMC11276507; doi:10.3390/curroncol31070285)
Supplement: Supplementary file 1 [file curroncol-31-00285-s001.zip › curroncol-3071267-supplementary.pdf]

Supplementary File

Supplementary Table S1: OPCS-4 codes used to screen for type of surgery.

Surgery	NCRAS (OPCS-4 codes)	SEER Codes
Appendicectomy	H01 (H01.1, H01.2, H01.3, H01.8, H01.9<br><br>H02 (H02.1, H02.2, H02.3, H02.4, H02.8, H02.9)<br><br>H03 (H03.1, H03.2, H03.3, H03.8, H03.9)	Codes 20,27,29,30,32
Right hemicolectomy	H06(H06.1, H06.2, H06.3, H06.4, H06.5, H06.8, H06.9)<br><br>H07 (H07.1, H07.2, H07.3, H07.4, H07.5, H07.8, H07.9)<br><br>H11 (H11.2, H11.3, H11.4, H11.5, H11.6, H07.8, H07.9- excluding H11.1)	Codes 40,41

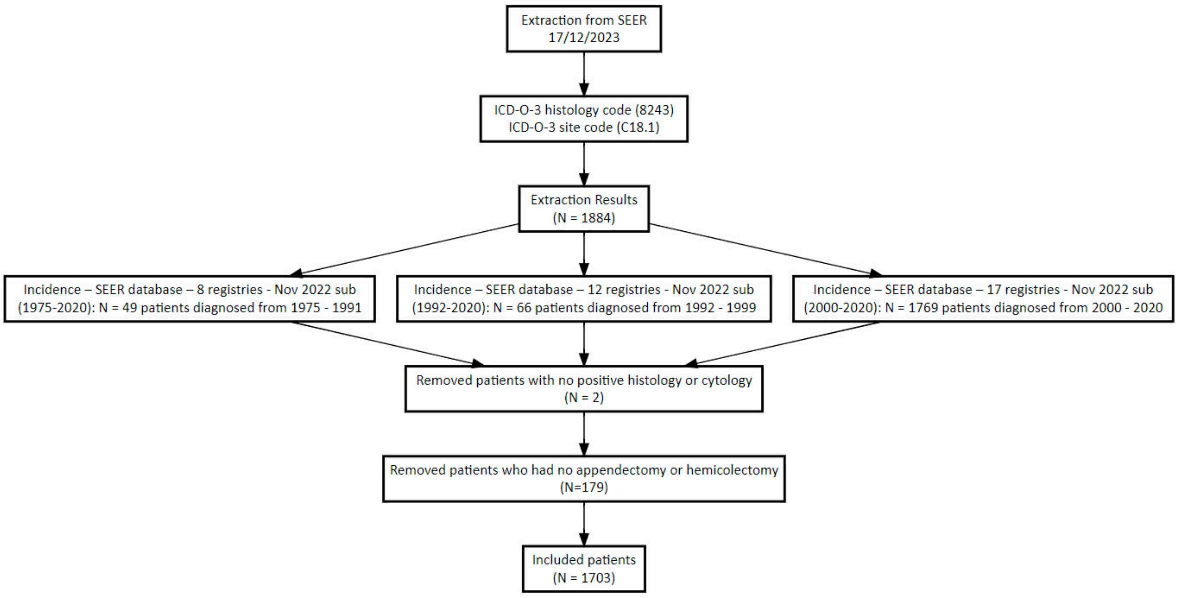

Supplementary Figure S1: Flowchart detailing stages of data extraction (SEER)

Supplementary Table S2: Variable formation (SEER)

Merged Variable	Original variables
Stage	• Combined Summary Stage (2004+)<br>• SEER historic stage A (1973-2015)
Tumour size	• EOD 4 - size (1983-1987)<br>• EOD 10 - size (1988-2003)<br>• CS tumor size (2004-2015)<br>• Tumor Size Summary (2016+)
Surgery type	• RX Summ--Surg Prim Site (1998+)<br>• Site specific surgery (1973-1997 varying detail by year and site)

Supplementary Table S3: Distribution of GCA cases across geographical regions in England (1995-2018)

	N	%
**Total**	1225	100
**Procedure**		
RHC	710	57.9
Appendicectomy	289	23.6
Neither	156	12.7
No procedure codes	70	5.7
**GOR**		
East Midlands	123	10.0
East of England	161	13.1
London	116	9.5
North East	60	4.9
North West	184	15.0
South East	176	14.4
South West	135	11.0
West Midlands	140	11.4
Yorkshire and The Humber	130	10.6

Supplementary Table S4: Uptake of right hemicolectomy procedures across geographical regions in England

GOR	N RHC	No RHC	N GCA	Uptake of RHC (%)	p-value
					**<0.001***
London	78	38	116	67.2	
North East	39	21	60	65.0	
East Midlands	79	44	123	64.2	
West Midlands	88	52	140	62.9	
Yorkshire and The Humber	73	57	130	56.2	
East of England	90	71	161	55.9	
South West	75	60	135	55.6	
North West	98	86	184	53.3	
South East	90	86	176	51.1	

Supplementary Table S5: Overall survival at 1 month, 12 months, 36 months, and 60 months

Data	Subgroup	1 month	12 months	36 months	60 months
**NCRAS**	**Overall**	98.0% (97.1-98.9%)	92.8% (91.2-94.4%)	81.0% (78.5-83.5%)	73.8% (70.9-76.7%)
**SEER**	**Overall**	98.6% (98.0 – 99.1%)	95.0% (93.9 – 96.1%)	87.2% (85.5 - 88.9%)	79.6 (77.4 - 81.8%)
**NCRAS**	**Appendicectomy**	98.6% (97.3-100%)	92.4% (89.4-95.5%)	77.9% (73.1-83.0%)	72.3% (67.0-78.1%)
	**RHC**	97.7% (96.7-98.8%)	92.9% (91.1-94.8%)	82.2% (79.4-85.1%)	74.3% (71.0-77.8%)
**SEER**	**Appendicectomy**	97.8% (96.8-98.9%)	94.2% (92.6-95.9%)	86.8% (84.3-89.4%)	79.5% (76.5-82.7%)
	**RHC**	99.2% (98.7 - 99.8%)	95.7% (94.3-97.1%)	87.5% (85.2-89.9%)	79.6% (76.6-82.8%)

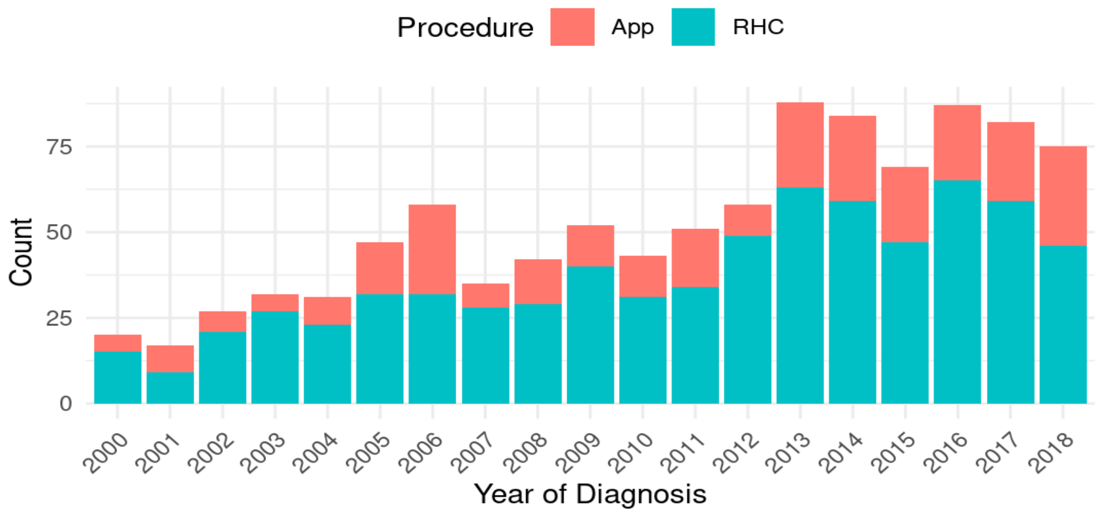

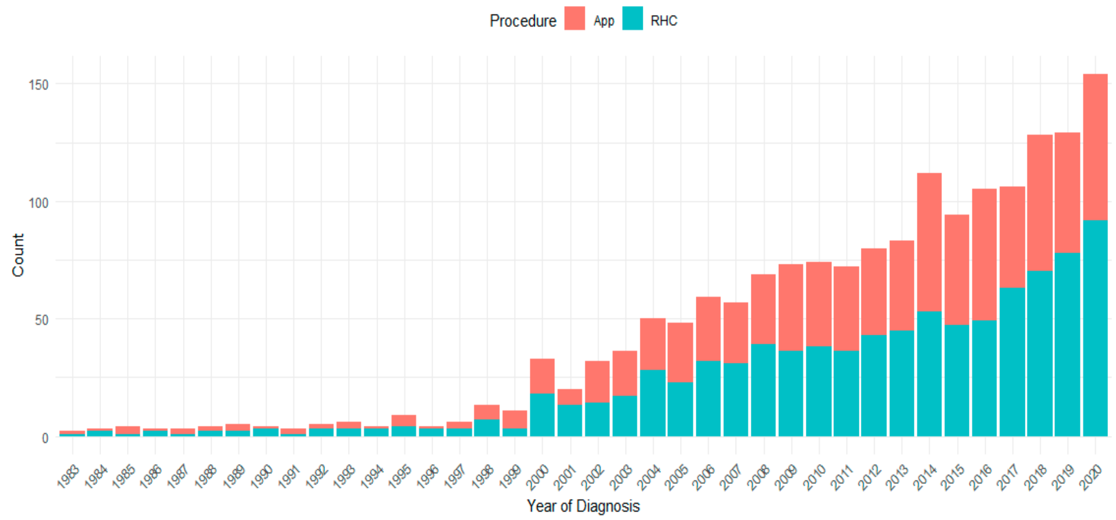

Supplementary Figure S2A (top): Distribution of procedure types by year of diagnosis (NCRAS)

Supplementary Figure S2B (bottom): Distribution of procedure types by year of diagnosis (SEER)
